# Subtype Differences in the Interaction of HIV-1 Matrix with Calmodulin: Implications for Biological Functions

**DOI:** 10.3390/biom11091294

**Published:** 2021-08-31

**Authors:** Alexej Dick, Simon Cocklin

**Affiliations:** Department of Biochemistry & Molecular Biology, Drexel University College of Medicine, Rooms 10307, 10309, and 10315, 245 North 15th Street, Philadelphia, PA 19102, USA

**Keywords:** HIV-1 matrix protein, human calmodulin-1, surface plasmon resonance, electrostatic complementarity

## Abstract

The HIV-1 Gag polyprotein plays essential roles during the late stage of the HIV-1 replication cycle, and has recently been identified as a promising therapeutic target. The N-terminal portion of the HIV-1 Gag polyprotein encodes the myristoylated matrix (MA) protein, which functions in the trafficking of the structural proteins to the plasma membrane (PM) and facilitation of envelope incorporation into budding virus. Numerous host cell proteins interact with the MA portion of the Gag polyprotein during this process. One such factor is the ubiquitous calcium-binding protein calmodulin (CaM), which interacts preferentially with myristoylated proteins, thereby regulating cell physiology. The exact role of this interaction is poorly understood to date. Atomic resolution structures revealed the nature of the CaM-MA interaction for clade B isolates. In this study, we expanded our knowledge and characterized biophysically and computationally the CaM interaction with MA from other HIV-1 clades and discovered differences in the CaM recognition as compared to the prototypical clade B MA, with significant alterations in the interaction with the MA protein from clade C. Structural investigation and in silico mutational analysis revealed that HIV-1 MA protein from clade C, which is responsible for the majority of global HIV-1 infections, interacts with lower affinity and altered kinetics as compared to the canonical clade B. This finding may have implications for additional altered interaction networks as compared to the well-studied clade B. Our analysis highlights the importance of expanding investigations of virus-host cell factor interaction networks to other HIV-1 clades.

## 1. Introduction

AIDS (Acquired Immune Deficiency Syndrome) is a global epidemic caused by two related lentiviruses, HIV-1 and the less pathogenic HIV-2 [1]. At the end of 2019, 38 million people lived with HIV worldwide, with 1.7 million newly infected and 690.000 AIDS-related deaths [2]. These statistics highlight the continued need for novel antiviral agents and new therapeutic targets to combat acquired and naturally occurring resistance and the toxicity problems of current therapeutics. One such emerging therapeutic target is the HIV-1 Gag protein and its component domains [3]. The HIV-1 Gag protein is the master regulator of co-factor packaging, assembly, and release of the immature virion [4,5]. Considerable effort has been expended upon targeting the HIV-1 CA protein, the nucleocapsid, as well as late domains (p6), and only a few studies have looked at the potential of the HIV-1 MA protein (reviewed in [3]).

The HIV-1 matrix (MA) protein is encoded as the N-terminal portion of the Gag polyprotein and has established roles in viral assembly. This small, multifunctional protein is responsible for directing the viral and cellular components to the assembly site and regulating the incorporation of the envelope glycoproteins into the budding virus [6,7,8]. The association of the Gag protein to the plasma membrane (PM) is highly dependent on the exposure of a myristoyl group (myr) and a basic patch at the N-terminus of the MA protein. The basic MA region has been demonstrated to interact with PIP_2_ or other PM lipids such as phosphatidylserine, phosphatidylcholine, and phosphatidylethanolamine [9]. The basic patch also has nucleic acid binding properties, and it is thought that the interplay between lipid and RNA binding is critical for specific interaction with only the plasma membrane [10,11,12]. The trafficking of Gag to the PM has been demonstrated to be facilitated by its interaction with several host cell proteins, including Arf and GGA proteins [13]. Additional interactions have been inferred, but their exact role is only poorly understood to date, including the human adaptor protein complexes AP-1, AP-2, and AP-3 [14,15,16], TIP47 [17], suppressor of cytokine signaling 1 (SOCS1) [18], a lymphoid specific Src kinase (Lck) [19], N-ethylmaleimide-sensitive factor attachment protein receptors (SNAREs) [20], Filamin A [21], vacuolar protein sorting-associated protein 18 (Vps18) [22], Lyric [23], Mon2 [22], and during virion budding possibly with the cellular endosomal complex required for transport (ESCRT) proteins such as ALIX [24]. Calmodulin is probably the best-studied HIV-1 MA interaction partner; however, these insights are mainly based on studies with HIV-1 clade B, and so far, few insights have been gleaned for its biological role in the context of the MA protein.

The HIV-1 MA protein has emerged as an attractive, if not elusive, antiviral target because of its fundamental roles in virion assembly and the high degree of conservation of its phosphoinositide 4,5-bisphosphate (PI[4,5]P_2_)/nucleic acid binding site [10,11,12,25]. The genetic diversity of HIV-1 is a major hurdle for both vaccine and drug discovery efforts. The impact of subtype-specific differences for agents targeting Env and RT is the most understood, but this problem eventually becomes relevant for all established and new therapeutic targets. As Gag and its component domains, including MA, are now being explored as viable therapeutic targets, it is imperative to understand the potential of these sometimes large changes at the sequence level on our ability to broadly inhibit their functions across clades. 

To date, all of the structural data and a great majority of the virological information on Gag and MA has been obtained by the study of one laboratory-adapted clade B isolate, HIV-1 (NL4-3). HIV-1 can differ by up to 10% in one infected individual, up to 35% in one subtype, and up to 50% between subtypes [26,27,28]. Studies looking at the impact of the sequence variability of the MA protein are sorely needed, given its new role as a therapeutic target and the fact that second only to Env, it is the most variable part of the HIV-1 genome [29]. Therefore, in this study, we aimed to take the first step in addressing the shortage of information on the biology, structures, and binding interactions of MA proteins from isolates from subtypes other than B, using the well-established calmodulin (CaM) interaction as a model system. To achieve this, we cloned, overproduced, and purified the MA proteins from HIV-1 clade A1 (92UG037.1), A2 (CDKTB48), B (LAI and NL4-3), C (92BR025.8), and D (94UG114.1) and biophysically characterized the kinetic- and affinity profiles of binding to human CaM-1 using surface plasmon resonance spectroscopy (SPR). We identified subtype-specific differences in hCaM-1 recognition, as judged by differential affinities. This was especially pronounced in the MA protein derived from our clade C isolate. To understand these differences at the molecular level, we generated a homology model and undertook rigorous conservation and electrostatic complementarity analysis. We collectively found that hCaM-1 interaction in vitro differs between clades with clade C MA displaying the lowest affinity towards hCaM-1 accompanied by a decreased on-rate compared to other clades. Through electrostatic complementarity analysis, we try to understand these alterations on a molecular level. We believe that those alterations observed in hCaM-1 recognition might also be echoed in the recognition of other host cell factors by the MA protein. As we observe differential interaction affinity and kinetics with CaM, which has a relatively conserved binding site, such subtype-specific differences in the interactions of other host cell factors may be more pronounced, depending upon the location and conservation of the binding site within the HIV-1 MA protein. A more rigorous evaluation across different HIV-1 clades, in addition to evaluating the effects upon potential ternary complexes, could increase our understanding of the role of CaM in HIV infection and its life cycle.

## 2. Material and Methods

### 2.1. Protein Expression and Purification

The MA domain of the HIV-1 gag gene was amplified from plasmid pLAI (a generous gift from Drs. Evelyn Kilareski and Brian Wigdahl, both Drexel University College of Medicine, Philadelphia, USA) using primers designed to facilitate ligation-independent cloning into the vector pETHSUL [30]. This vector is designed for the insertion of genes of interest in frame with an N-terminal small ubiquitin-related modifier (SUMO) tag. Clade A1 (92UG037.1), A2 (CDKTB48), B (LAI and NL4-3), C (92BR025.8), and D (94UG114.1) and human Calmodulin-1 (hCaM-1, GenBank accession no. AAA35635.1) genes were synthesized from Genescript Corp. (Piscataway, NJ, USA). The recombinant pETHSUL plasmid was verified for the presence of MA or hCaM-1 insert by restriction digestion and sequence analysis (Genewiz, Inc., South Plainfield, NJ, USA). The Escherichia coli strain BL21 (DE3) Codon+-RIL (Stratagene, La Jolla, CA, USA) was used for the expression of H6 pSUMO-MA. Luria-Bertani broth (4 mL) containing 100 mg/mL ampicillin and 50 mg/mL chloramphenicol was inoculated with a single transformed colony, and the colony was allowed to grow at 37 °C for overnight. A 1 mL aliquot of the preculture was used to inoculate 1000 mL of the autoinducing media ZYP-5052 [31]. The culture was grown at 30 °C for 16 h. Cells were harvested by centrifugation at 5000 rpm for 20 min at 4 °C, and the pellet was resuspended in 20 mM Tris HCl pH 8.0, 1 M NaCl, 2 M LiCl, 2 mM Imidazole (20 mM Tris HCl pH 7.5, 1M NaCl, 10 mM CaCl_2_, 2 mM Imidazole for hCaM-1). Cells were lysed by sonication, and the supernatant was clarified by centrifugation at 40,000 rpm (Ti45i, Beckmann) for 45 min at 4 °C. The supernatant was removed and applied to a 5 mL TALON cobalt resin affinity column (ClonTech), previously equilibrated with resuspension buffer. The bound supernatant was washed for 100 mL with wash buffer 1 (MA: 20 mM Tris HCl pH 9.0, 1 M NaCl, 2 mM ATP, 4 mM Imidazole; hCaM-1: 20 mM Tris HCl pH 7.5, 1 M NaCl, 2 mM ATP, 10 mM CaCl_2_, 4 mM Imidazole) and 100 mL wash buffer 2 (MA: 20 mM Tris HCl pH 8.0, 400 mM NaCl, 8 mM Imidazole; hCaM-1: 20 mM Tris HCl pH 7.5, 400 mM NaCl, 10 mM CaCl_2_, 8 mM Imidazole). Tightly associated proteins were eluted in six-column volumes of 20 mM Tris HCl pH 8.0, 400 mM NaCl, 250 mM Imidazole. To this pooled sample, 10 µg of a recombinant His6-tagged form of the catalytic domain (dtUD1) of the Saccharomyces cerevisiae SUMO hydrolase was added [30]. Cleavage was allowed to proceed for 2 h at 25 °C. Following cleavage, the sample was dialyzed at 4 °C overnight against 2 L of 20 mM Tris HCl pH 8.0, 150 mM NaCl for MA, or against 20 mM Tris HCl pH 8.0, 150 mM NaCl, 10 mM CaCl_2_ for hCaM-1 to remove any imidazole. After dialysis, the hCaM-1 dtUD1-catalyzed cleavage reaction was concentrated and subjected to a Sephacryl S-100 16/60 column with a downstream 5 mL Hi-Trap Ni-NTA (GE Healthcare, Chalfont, UK) column equilibrated with dialysis buffer to remove dtUD1. HCaM-1 was pooled, concentrated, flash-frozen in liquid nitrogen, and stored at −80 °C. Dialyzed MA was applied to a second TALON cobalt resin affinity column. In this purification step, however, the cleaved MA/hCaM-1 protein passes straight through the column owing to the removal of the His6 tag. Subsequently, the subtractively purified MA was dialyzed overnight at 4 °C against 25 mM Tris-HCl, pH 8.0, containing 10% glycerol. This dialyzed sample was then filtered and loaded onto a 5 mL Hi-Trap Q HP column (GE Healthcare, Chalfont, UK). The flowthrough, containing the MA protein, was concentrated, flash-frozen in liquid nitrogen, and stored at −80 °C.

### 2.2. SPR Direct Interaction Analysis

All binding assays were performed on a ProteOn XPR36 SPR Protein Interaction Array System (Bio-Rad Laboratories, Hercules, CA, USA). The instrument temperature was set at 25 °C for all kinetic analyses. ProteOn GLH sensor chips were preconditioned with two short pulses each (10 s) of 50 mM NaOH, 100 mM HCl, and 0.5% sodium dodecyl sulfide. Then the system was equilibrated with buffer A (50 mM Tris pH 7.0, 100 mM NaCl, 5 mM CaCl_2_, and 0.005% polysorbate 20) or B (50 mM Tris pH 7.0, 100 mM NaCl, 10 mM EDTA, and 0.005% polysorbate 20). The surface of a GLH sensor chip was activated with a 1:100 dilution of a 1:1 mixture of 1-ethyl-3-(3-dimethylaminopropyl) carbodiimide hydrochloride (0.2 M) and sulfo-N-hydroxysuccinimide (0.05 M). Immediately after chip activation, the HIV-1 MA protein constructs were prepared at a concentration of 10 μg/mL in 10 mM sodium acetate, pH 5.5, and injected across ligand flow channels for 5 min at a flow rate of 30 µL/min. Then, after unreacted protein had been washed out, excess active ester groups on the sensor surface were capped by a 5 min injection of 1M ethanolamine HCl (pH 8.0) at a flow rate of 5 μL/min. A reference surface was similarly created by immobilizing a nonspecific protein (IgG b12 anti-HIV-1 gp120; was obtained through the NIH AIDS Reagent Program, Division of AIDS, NIAID, NIH: Anti-HIV-1 gp120 Monoclonal (IgG1 b12) from Dr. Dennis Burton and Carlos Barbas) and was used as a background to correct nonspecific binding. Serial dilutions were then prepared in the running buffer A or B and injected at a flow rate of 100 µL/min, for a 50 s association phase, followed by up to a 5 min dissociation phase using the “one-shot kinetics” capability of the ProteOn instrument [32]. Data were analyzed using the ProteOn Manager Software version 3.0 (Bio-Rad). The responses from the reference flow cell were subtracted to account for the nonspecific binding and injection artifacts. Experimental data were fitted to a simple 1:1 binding model. The average kinetic parameters (association [k_on_] and dissociation [k_off_] rates) generated from 3 data sets were used to define the equilibrium dissociation constant (K_D_).

### 2.3. In Silico Homology Modeling, Optimization, and Conservation Analysis

Homology modeling of the HIV-1 MA protein from clade C (92BR025.8) was performed using the swiss-model server, the amino acid sequence of HIV-1 MA (NL4-3) as a reference, and the crystal structure of 1HIW as a template. Multiple sequence alignment was performed with the Clustal Omega server [33], and the resulting alignment was manually adjusted using BioEdit. Optimization of the models was achieved using energy minimization protocols within YASARA [34]. The homology model was further improved with MOLProbity, including placement of hydrogens, Asn/Gln/His flips, and all-atom contacts and geometry evaluated [35]. Ramachandran plots for the models were assessed, and Ramachandran outlier residues were fixed with COOT [36] and energy minimized. The optimized model was subjected to conservation analysis using the ConSurf server [37] or the APBS [38] plugin for electrostatic calculations within PyMOL. High-resolution figures were prepared using PyMOL. For the electrostatic complementarity calculations, Flare version 4 was used (Cresset^®^, Litlington, Cambridgeshire, UK).

## 3. Results

### 3.1. HIV-1 MA Conservation within the CaM Binding Motif

**Figure 1:**
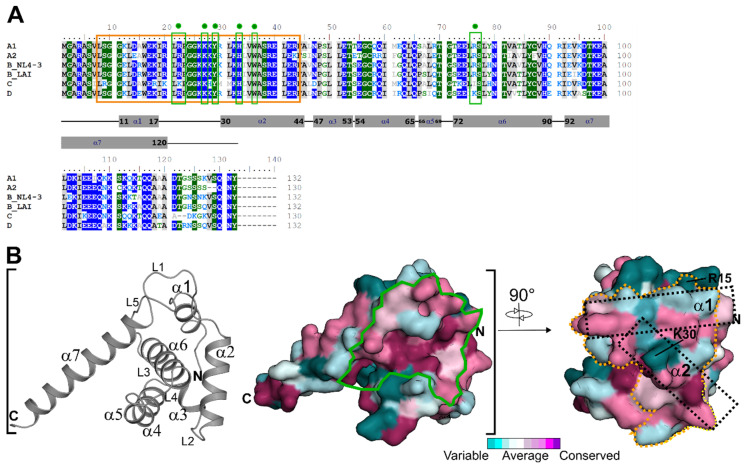
Multiple sequence alignment of HIV-1 MA clades used in this study and evolutionary conservation. (**A**) Amino acid sequences of HIV-1 MA protein from clade A1 (92UG037.1, GenBank accession no. AAC97550.1), A2 (CDKTB48, GenBank accession no. AF286238.1), B (NL4-3, GeneBank accession no. AF003887.1), C (92BR025.8, GenBank accession no. U52953.1), D (94UG114.1, GenBank accession no. U88824.1) were aligned using Clustal Omega [33] and manually adjusted in BioEdit. Residues with greater than 70% conservation are color-shaded (hydrophilic residues are in blue, neutral in green, and hydrophobic in grey). The yellow box insert highlights residue 8-43, the CaM binding motif. The green box inserts with a circle highlight the residues involved in di-C4-PI(4,5)P_2_ binding. Sequence identity in comparison to HIV-1 clade B (NL4-3): A1 (92UG037.1): 82.6%, A2 (CDKTB48): 78.8%, B(LAI): 93.2%, C (92BR025.8): 72.0% (68.3% within residue 8-43), and D (94UG114.1): 81.1%. (**B**) Secondary structure (loop: L, α: alpha-helix) and conservation analysis of the HIV-1 MA protein based on the MSA in panel A. Conservation is represented on the surface of HIV-1 MA from clade B (PDB code: 1HIW). The solid green line indicates the PI[4,5]P2/nucleic acid binding site, while the dotted orange line indicates the CaM binding motif. Dotted black rectangles highlight helix α-1 and α-2 within the CaM binding motif.

The N-terminal region of HIV-1 MA is crucial for multiple HIV-1 Gag functions, including membrane interaction via myristate exposure, binding to several host factors to facilitate Gag trafficking, and gp160 incorporation [9,39,40,41,42]. Therefore, we generated a multiple sequence alignment (MSA) of the here studied clades (A1 (92UG037.1), A2 (CDKTB48), B(NL4-3), B(LAI), C (92BR025.8), and D (94UG114.1)) to identify critical residues and differences within the studied clades in hCaM-1 recognition. The MSA indicates high conservation in crucial regions such as the PI[4,5]P_2_/nucleic acid binding site (Figure 1A,B, solid green line). However, if compared to clade B (NL4-3), the overall conservation differs within the studied clades. Most noticeably, clade C displays the lowest conservation and a sequence identity of 72.0% compared to clade B (NL4-3). 

Interestingly, also the minimal CaM binding motif of HIV-1 MA residue 8-43 [43] (Figure 1B, yellow dotted line) displays high conservation; however, clade C again with the lowest sequence identity of 68.3%. The solution NMR complex structure indicates major hydrophobic interactions of HIV-1 MA helix α1 and α2 with the C- and N-terminal lobe of rnCaM (rn—Rattus norvegicus) [43]. These residues in helix α1 are Leu13, Trp16, Ile19, and Leu21 (interact with C-terminus of rnCaM) and helix α2 with Tyr29, Ile34, Val35, Ala37, and Leu41 (interact with the N-terminus of rnCaM) are present in all clades (Figure 1A). Noticeably, Leu31 is replaced by Met31 in clade C. Besides those similarities, there are noticeable changes in clade C. The most prominent changes are Arg9 (Ser in all other clades), Ala15 (Arg in clade B, Glu in clade D, Ala in clade A1 and A2), His28 (Lys in all other clades), and Met30 (Lys/Arg in all other clades).

### 3.2. Differential Recognition of Human CaM-1 by HIV-1 MA Proteins

As we saw apparent differences at the sequence level between clades throughout the protein and in the CaM binding motif, we decided to investigate if those differences might affect hCaM-1 recognition. Numerous biophysical and biochemical works have been performed on the CaM-HIV-1 MA interaction during recent years [43,44,45,46]. However, the precise role of this interaction in the viral life-cycle remains elusive. Moreover, all studies utilized clade B, laboratory-adapted isolate HIV-1 (NL4-3). To extend our knowledge to other subtypes, other than subtype B (NL4-3) using the CaM interaction as a model, we cloned, expressed, and purified full-length HIV-1 MA clade A1 (92UG037.1), A2 (CDKTB48), B(NL4-3), B(LAI), C (92BR025.8), and D (94UG114.1) (Figure S1) to shed light on possible alterations in CaM recognition using surface plasmin resonance interaction analysis.

**Figure 2:**
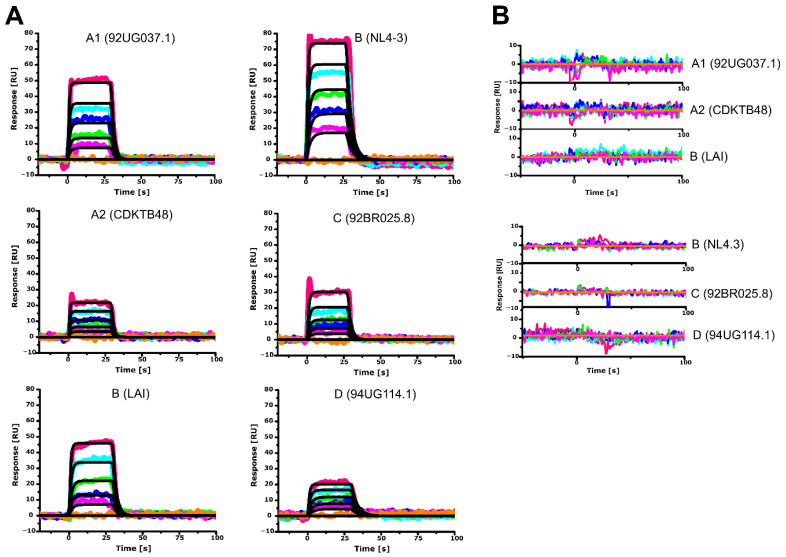
Sensorgrams depicting the interaction of hCaM-1 with immobilized clades of the HIV-1 MA protein. Human CaM-1 concentrations of 0, 6.25, 12.5, 25, 50, and 100 µM were tested and represented as orange, magenta, green, blue, cyan, and red lines. Colored lines indicate experimental data, whereas black lines indicate fitting to a simple 1:1 binding model. (**A**) In the presence of 5 mM CaCl_2_ or (**B**) in the presence of 10 mM EDTA. Colored lines represent actual data, whereas black lines indicate fitting to a 1:1 interaction model. Experiments were performed in triplicate.

CaM performs its function in a Ca^2+^ dependent manner (reviewed in [47]). We reproduced the Ca^2+^ dependence of all the here studied HIV-1 MA clades as shown by the sensorgrams in Figure 2A,B. As expected, removal of Ca^2+^ and addition of 10 mM EDTA completely disrupted hCaM-1 binding to all HIV-1 MA clades (Figure 2B). For the first time to our knowledge, we also show that HIV-1 MA clade A1 (92UG037.1), A2 (CDKTB48), B(NL4-3), B(LAI), C (92BR025.8), and D (94UG114.1) bind hCaM-1 in a Ca^2+^ dependent manner (Figure 2A,B). All clades showed, as expected, no detectable response in the absence of Ca^2+.^

**Figure 3:**
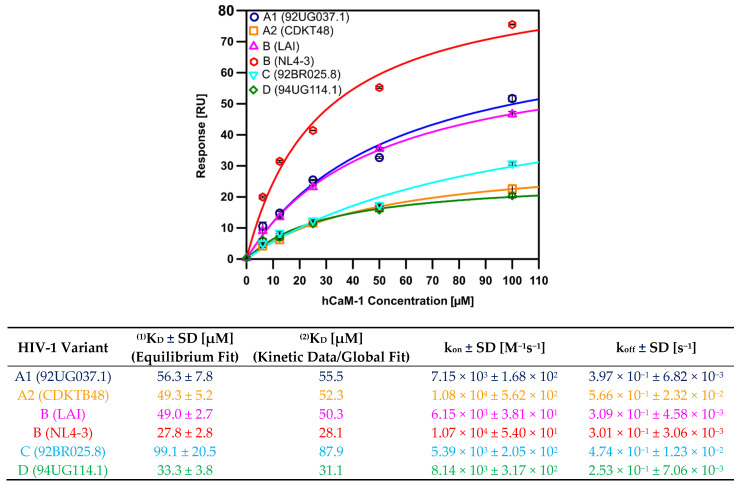
Binding isotherms of hCaM-1 to immobilized full-length HIV-1 MA from clade A1, A2, B, C, and D, and the corresponding kinetic parameters in the table below. Binding isotherms are derived from the data in Figure 2. Experiments were performed in triplicate and data displayed with standard deviations (SD). ^(1)^ Equilibrium Dissociation Constant (K_D_) derived from a Langmuir isotherm equilibrium fit or ^(2)^ from a global fit and the derived kinetic data. A1 (92UG037.1), A2 (CDKTB48), B(NL4-3), B(LAI), C (92BR025.8), and D (94UG114.1).

We next fitted the experimental data to a simple 1:1 binding model to determine the equilibrium dissociation constant (K_D_) of those interactions (Figure 3). All tested clades showed affinities in the low µM range, with clade B (NL4-3) displaying the highest affinity for hCaM-1 with a K_D_ value of 27.6 ± 5.6 µM. Notably, within the same clade, however, LAI showed a reduced affinity for hCaM with a K_D_ of 49.0 ± 2.7 µM, an almost two-fold reduction compared to NL4.3. This is most likely due to the slower on-rate of LAI (6.15 × 10^3^ ± 3.81 × 10^1^ M^−1^s^−1^) compared to NL4.3 (1.07 × 10^4^ ± 5.40 × 10^1^ M^−1^s^−1^). In line with clade B, HIV-1 MA protein from clade D (94UG114.1) displayed similar affinities (33.3 ± 3.8 µM) for hCaM primarily driven by the slowest off-rate of all the studied clades (2.53 × 10^−1^ ± 7.06 × 10^−3^ s^−1^). Clade A2 (CDKTB48) and B (LAI) share similar affinities for hCaM; however, their kinetic profile is reciprocal to each other’s kinetic profile, offsetting each other and therefore displaying similar K_D_. 

Interestingly, clade C (92BR025.8), responsible for 48% of infections worldwide, displayed a 3.5-fold reduced affinity towards hCaM-1 in comparison to clade B (NL4-3) and a 2-fold difference compared to the other subtype B isolate used in this study, LAI. Clade C (92BR025.8) also showed a two-fold slower on-rate and a faster off-rate as compared to NL4-3 (Figure 3 right table). We noticed that the faster off-rates, especially for clade C (92BR025.8) and A2 (CDKTB48), are primarily responsible for the reduced affinities for hCaM. Both clades have noticeable amino acid alterations within the CaM binding motif (Figure 1A), with significant alterations for clade C (92BR025.8). Recent biophysical and biochemical studies showed that the N-terminal region of HIV-1 MA, including residues 8-43, which makes up helix α1, a connecting basic loop, and helix α2 is crucial for this interaction with CaM. To exclude the involvement of the C-terminal region of HIV-1 MA, we generated for clade A1 (92UG037.1) and C (92BR025.8), the clades with the most significant affinity decrease, constructs lacking the C-terminal helix α5 (construct with residue 1-109). Both truncated constructs from clade A1 (92UG037.1) and C (92BR025.8) showed similar binding characteristics compared to their full-length counterparts (Figure S2). Based on the equilibrium and kinetic data and the most prominent effect seen for clade C (92BR025.8) MA protein, we decided to investigate the molecular details that could explain this altered binding characteristic focusing on clade C (92BR025.8) and clade B (NL4.3).

### 3.3. Structural and Electrostatic Alterations between HIV-1 Subtypes

Having biophysically quantified the recognition of hCaM-1 by the different variants of the HIV-1 MA protein and identified that the clade C (92BR025.8) out of all the studied variants displayed the most significant affinity reduction, we looked into possible molecular explanations for our observations. To understand the reduced binding affinity of HIV-1 MA (92BR025.8) from clade C to hCaM-1, we next generated a homology model of HIV-1 MA (92BR025.8) based on the crystal structure of HIV-1 MA from clade B (NL4-3). The solution complex structure of HIV-1 MA (NL4-3) bound to rnCaM solved by NMR [43] showed that the CaM-binding interface consists of hydrophobic and electrostatic interactions, mainly basic in nature. 

The overall homology structure of the HIV-1 MA (92BR025.8) is very similar to HIV-1 MA (NL4-3) with root-mean-square deviations (rmsd) of 0.29 Å over 115 Cα atoms. The HIV-1 MA (92BR025.8) structure is mainly alpha-helical, starting with helix α1 and α2 at the N-terminus, connected via a basic loop that represents the CaM binding motif. The MA core contains the highly positive PI[4,5]P_2_/nucleic acid binding site made up of helix α2, helix α5, and helix α6. This arrangement is also present in our homology model of HIV-1 (92BR025.8). 

**Figure 4:**
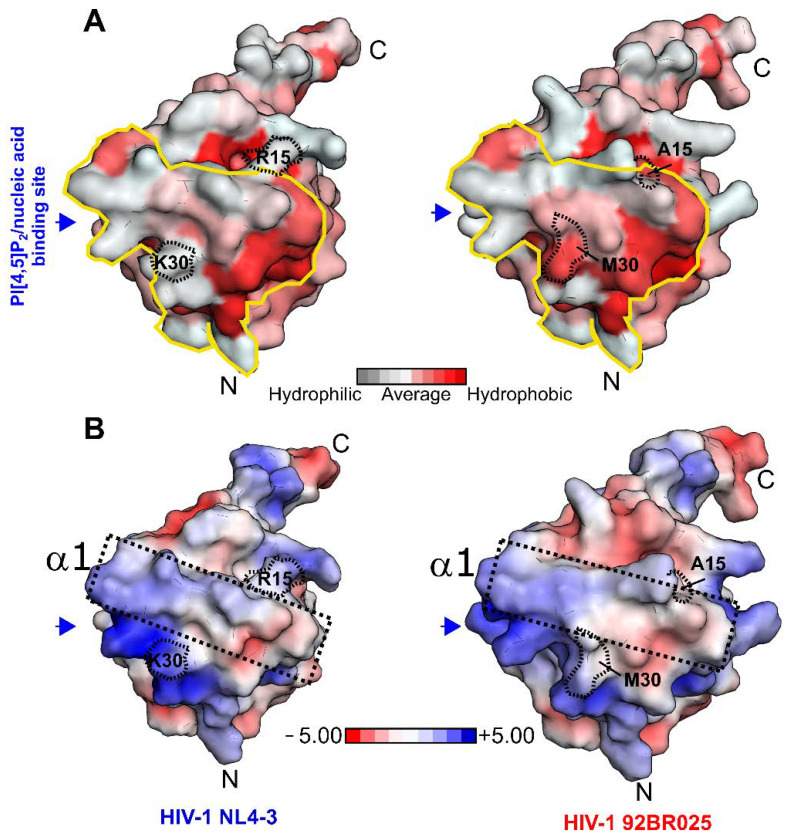
Surface conservation plot and electrostatic surface potential of the HIV-1 MA protein. (**A**) Hydrophobicity plot mapped on the solvent-accessible surface of the HIV-1 MA (NL4-3) structure (left panel, PDB code: 1HIW) and in this study generated homology model of clade C (92BR025.8, right panel) [48]. (**B**) The electrostatic surface potential of HIV-1 MA clade B (NL4-3) (left panel, PDB code: 1HIW) and in this study generated homology model of clade C (92,BR025.8, right panel). N- and C-termini are highlighted with an N or C, respectively. Box highlights helix α1 and loop region between α1 and α2. The electrostatic potential is shown between −5 kcal/mol*e (red) to +5 kcal/mol*e (blue). The electrostatic surface calculation was performed at 310K and 150 mM Salt concentration with APBS [38]. The blue arrow indicates the (PI[4,5]P2)/nucleic acid binding site and the solid yellow line the CaM binding region.

We next compared the solvent-exposed hydrophobic characteristics as well as the electrostatic surface potential with the HIV-1 MA (NL4-3) structure (PDB code: 1HIW) (Figure 4A,B). Interestingly, although HIV-1 MA (92BR025.8) displays a reduced hCaM-1 affinity, the hydrophobic character of the CaM-binding region is, to some extent, increased by the introduction of Ala15 and Met30 (Figure 4A). In the HIV-1 MA (NL4-3) structure, helix α1 is predominantly basic in nature together with the subsequent loop (upstream to helix α2) (Figure 4B, black dotted box). After visual inspection, this solvent-exposed basic character is most likely reduced in HIV-1 clade C (92BR025.8) by introducing Ala15, His28, and Met30 (Figure 4B, black dotted box). We, therefore, believe that not exclusively but to a certain extent, this alteration might contribute to the reduced recognition of hCaM-1 by the HIV-1 MA (92BR025.8) protein from clade C. 

### 3.4. Electrostatic Complementarity of the HIV-1 MA/rnCaM Complex 

**Figure 5:**
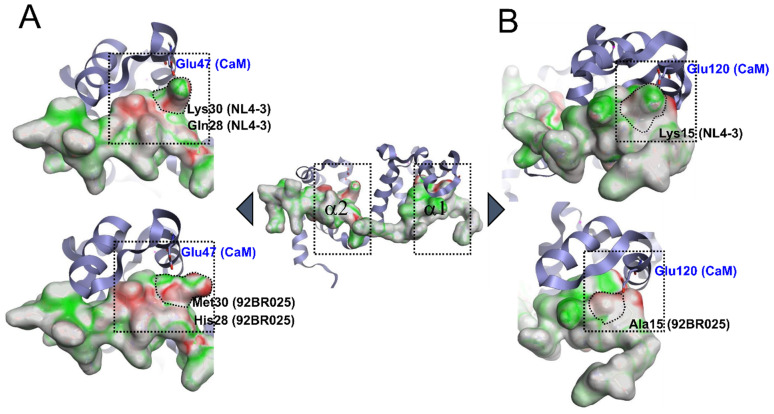
Electrostatic complementarity (EC) of the CaM binding motif of HIV-1 MA (residue 8-43) bound to rnCaM (PDB code: 2MGU). (**A**) Native complex and an (**B**) in silico mutated and minimized HIV-1 MA. Regions of the HIV-1 MA surface where there is perfect electrostatic complementarity with the protein are colored green, while the regions where there is a perfect electrostatic clash are colored red. RnCaM is displayed in ribbon, and HIV-1 MA (residue 8-43) is surface representation. Mutations that altered electrostatic complementarity and abrogated salt bridge interactions to Glutamates are outlined (Lys30Met, Lys15Ala). HIV-1 MA (8-43) clade B (NL4-3) complementarity score: 0.14; HIV-1 MA (8-43) clade C (92BR025.8) complementarity score: 0.12.

Having identified a slight increase in the hydrophobic surface area and reduction in the electrostatic potential by the presence of Ala15 and Met30 in the clade C(92BR025.8) MA protein, we sought further validation and quantify our hypothesis by calculating the electrostatic complementarity between the solution complex of HIV-1 MA (92BR025.8 vs. NL4-3) and rnCaM using Flare v4 (Cresset, Litlington, Cambridgeshire, UK). Electrostatic complementarity (EC) maps are based on a calculation of electrostatic potentials for the ligand (MA peptide residue 8-43) and the protein (rnCaM) on the surface of the ligand. These potentials are then added together, normalized, and scaled. Regions of the ligand with a score of 1 (meaning perfect electrostatic complementarity) are colored in green, a score of 0 will be colored in white, while the regions where there is a perfect electrostatic clash are colored in red with a score of −1. Flare v4 can also compute several electrostatic complementarity summary scores for quantification and comparison of the EC. Therefore, we performed this analysis with the native complex and an in silico mutated and minimized HIV-1 MA (Lys/Arg15Ala, Lys28His, Lys/Arg30Met), which mimics the clade C (92BR025.8) MA protein (Figure 5). In the reported solution structure, Glu47 of rnCaM forms a crucial salt bridge with Lys30 of HIV-1 MA of clade B (NL4-3). However, in our in silico mutated and minimized structure that resembles the clade C (92BR025.8) structure, Lys30 is mutated to a methionine. This mutation reduced the electrostatic complementarity towards rnCaM, as indicated by the increased red surface area (Figure 5A, compare upper and lower structure). More importantly, this mutation abrogates the crucial salt bridge with Lys30.

Furthermore, at the N-terminal region (Figure 5B) of HIV-1 MA clade B (NL4-3), Lys15 forms an additional salt bridge with Glu120 from rnCaM. In clade C (92BR025.8), this Lys15 is mutated to an alanine, the electrostatic complementarity reduced, and more importantly, the salt bridge abolished (Figure 5B, compare upper and lower structure). The overall complementarity score of the HIV-1 MA peptide (residue 8-43) is therefore also reduced, from 0.14 in clade B (NL4-3) to 0.12 in clade C (92BR025.8), indicating an overall reduction in the electrostatic complementarity. Taken together, the alterations in the clade C (92BR025.8) MA protein, increased hydrophobicity, and abolishment of crucial salt bridges by the introduction of Ala15 and Met30 could explain the reduced binding affinity accompanied by a slightly slower on-rate and faster off-rate in hCaM-1 recognition (Figure 2 and Figure 3).

## 4. Conclusions

The HIV-1 Gag polyprotein plays an important role during the late stage of the HIV-1 replication cycle, including trafficking to the PM and facilitation of envelope incorporation into the PM for subsequent virus assembly and budding. Numerous host cell proteins interact with the Gag portion during this process. The N-terminal portion of the Gag encodes the matrix protein, which is a driving force in PM association of Gag by binding specifically to PI(4,5)P_2_ and anchoring a hydrophobic myristoyl (myr) moiety into the PM. Exposure of the myr moiety is triggered by PI(4,5)P2 binding and by interacting with host cell factors [8,44,49,50]. One such factor is the ubiquitous calcium-binding protein calmodulin, expressed in all eukaryotic cells. CaM is known to interact with numerous targets, preferential to myristoylated proteins, thereby regulating cell physiology. HIV-1-infected cells show distinct subcellular distribution of CaM compared to uninfected cells. Previous studies revealed that CaM binds, in addition to Gag, also to the cytoplasmic tail (CT) of the gp160 protein, suggesting a role of CaM in the virus assembly step. The exact role of this function has yet to be elucidated. Small-angle x-ray scattering and NMR provided the molecular details of the CaM-MA interaction, which is mainly driven by hydrophobic interactions of the amphipathic helix α1 and α2 at the N-terminus of MA and the N- and C-terminal lobe of CaM. In addition to the hydrophobic interactions, several stabilizing salt bridges were identified. A minimal CaM binding peptide spanning residues 8-43 in the MA protein was shown to be essential for this interaction.

Previous and current structural work focuses mainly on the Western world circulating clade B (NL4-3) MA protein, including the molecular details for CaM recognition. In this study, we sought to expand our knowledge of the MA-CaM interaction and explore other HIV-1 MA clades, including A1 (92UG037.1), A2 (CDKTB48), C (92BR025.8), and D (94UG114.1). We successfully cloned and expressed these clades to biophysically characterize the interaction with human CaM-1 using surface plasmon resonance spectroscopy. As expected, binding of hCaM-1 for all studied clades was Ca^2+^-dependent with affinities in the mid micromolar range.

Interestingly, HIV-1 MA from clade C (92BR025.8) displayed the lowest affinity and reduced on-rate for hCaM-1 with a K_D_ value of 99.1 µM, a 3.5-fold reduction compared to clade B (NL4-3). 

To understand the possible structural features responsible for this altered hCaM-1 recognition, we generated a homology model of HIV-1 MA from clade C (92BR025.8) based on the crystal structure of clade B (NL4-3) MA protein. Our homology model showed when compared to clade B a similar, mainly alpha-helical topology, including helix α1 and α2, the main CaM binding motif at the N-terminus. 

Finally, using a combinational approach of sequence, electrostatic/hydrophobic surface potential, and electrostatic complementarity analysis, we could identify single amino acid alterations at the N-terminus that we believe could be responsible for the reduced affinity and on-rate of HIV-1 MA clades C (92BR025.8) for hCaM-1. Although the CaM binding to MA is favored by hydrophobic interactions as detailed by previous SAXS and NMR studies, additional electrostatic salt bridges, mainly Lys15 from MA with Glu120 from CaM and Lys30 from MA with Glu47 from CaM stabilize the CaM recognition. Both lysine residues are altered to alanine or methionine in the HIV-1 MA clade C (92BR025.8) protein, abrogating possibly essential stabilization of the initial formation of the CaM-MA complex as indicated by the reduced electrostatic complementarity within this mutated region. Therefore, our EC analysis could, to some extent, explain the here observed reduced affinity and altered kinetics of the HIV-1 MA clade C (92BR025.8) protein relative to clade B (NL4-3) MA protein. Cellular Ca^2+^-bound CaM or Apo-CaM make up to 0.1% of the total cellular proteins, and total cellular CaM concentrations are within 2–25 μM depending on tissue type [51,52]. Therefore, a reduced affinity and altered kinetics of hCaM to the HIV-1 MA clade C (92BR025.8) protein could have implications for myristoyl exposure from the N-terminal site of the MA protein as a function of MA unwinding kinetics within the CaM-MA complex and therefore MA-membrane interaction and subsequently Env incorporation. This Env incorporation might therefore be also clade-specific with modest mechanistic differences of Env incorporation. However, it is known that upon HIV-1 infection, CaM is overexpressed and cellular CaM concentrations increased; this, however, is dependent on already incorporated Env in the cellular membrane that provides a CaM interaction site at the C-terminus of the Env. Further studies are required to understand if, in the context of infected cells, CaM still has altered preferences for MA proteins from different clades, specifically to clade C.

Additionally, MA is bound to cellular RNA during translocation to the membrane, where RNA serves as a chaperon to prevent MA binding to inappropriate membrane compartments containing mainly phosphatidylserine (PS) and phosphatidylcholine (PC) lipids. However, the high concentration of PI(4,5)P2 acidic lipids within the plasma membrane results in RNA dissociation and MA-membrane binding [53,54]. The RNA and CaM binding sites in MA partly overlap, and CaM interaction with MA could have an effect on the kinetics of RNA dissociation and membrane binding of MA. If a clade-specific effect on membrane binding resulting from altered CaM recognition is present in vivo, it has to be further investigated.

In summary, the MA-CaM interaction, thought to be similar across HIV-1 clades, has, according to our biophysical and computational analysis, more nuanced features than initially thought. We showed for the first time to our knowledge that in HIV-1 clade C (92BR025.8), which is responsible for the majority of global HIV-1 infections, single point mutations in the CaM binding motif on the MA protein can alter hCaM-1 recognition with potential, yet still to be explored, consequences in vivo. These differences may also have implications for other MA-host protein interactions, especially those mediated by binding sites in more variable regions of the MA protein.

## Data Availability

Not applicable.

## Supplementary Materials

The following are available online at https://www.mdpi.com/article/10.3390/biom11091294/s1, Figure S1: Overexpressed and purified HIV-1 MA proteins from clade A1, A2, B (NL4-3 and LAI), C, and D. Figure S2: Direct binding of HIV-1 MA from clade A1 (92UG037.1) and C (92BR025.8) lacking residue 110-132 to hCaM-1. Experiments were performed in triplicate.
