# Supplementary material for: Subtype Differences in the Interaction of HIV-1 Matrix with Calmodulin: Implications for Biological Functions
Source: Biomolecules. 2021 Aug 31;11(9):1294. doi: 10.3390/biom11091294 (PMC8464830; doi:10.3390/biom11091294)
Supplement: Supplementary file 1 [file biomolecules-11-01294-s001.zip › biomolecules-1318968-supplementary.pdf]

Supplementary information

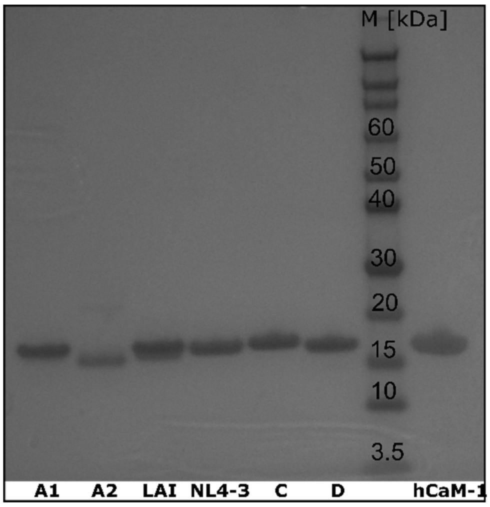

Supplementary Figure S1. Overexpressed and purified HIV-1 MA proteins from clade A1, A2, B (NL4-3 and LAI), C and D.

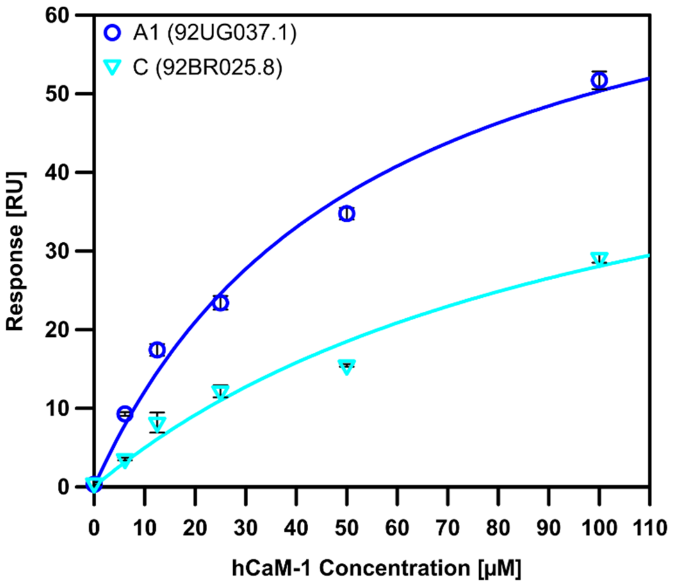

HIV-1 Variant (residue 1-109)	K_D [μM]	k_{on} [$M^{-1}s^{-1}$]	k_{off} [s^{-1}]
**A1 (92UG037.1)**	54.3 ± 6.3	$6.43 \times 10^3 \pm 2.64 \times 10^2$	$4.07 \times 10^{-1} \pm 1.19 \times 10^{-1}$
C (92BR025.8)	109.0 ± 28.7	$3.50 \times 10^3 \pm 1.95 \times 10^2$	$3.31 \times 10^{-1} \pm 1.08 \times 10^{-1}$

Supplementary Figure S2. Direct binding of HIV-1 MA from clade A1 (92UG037.1) and C (92BR025.8) lacking residue 110-132 to hCaM-1. Experiments were performed in triplicate.
